# Face masks affect perception of happy faces in deaf people

**DOI:** 10.1038/s41598-022-16138-x

**Published:** 2022-07-20

**Authors:** Maria Bianca Amadeo, Andrea Escelsior, Mario Amore, Gianluca Serafini, Beatriz Pereira da Silva, Monica Gori

**Affiliations:** 1 U-VIP Unit for Visually Impaired People, Fondazione Istituto Italiano di Tecnologia, Via Enrico Melen 83, 16152 Genoa, Italy; 2 Applied Neurosciences for Technological Advances in Rehabilitation Systems (ANTARES) Joint Lab, Clinica Psichiatrica Ed SPDC, Largo Rosanna Benzi, 10, 16132 Genoa, Italy; 3 Department of Neuroscience, Rehabilitation, Ophthalmology, Genetics, Maternal and Child Health (DINOGMI), Section of Psychiatry, University of Genoa, Genoa, Italy; 4 IRCCS Ospedale Policlinico San Martino, Genoa, Italy

**Keywords:** Psychology, Auditory system, Emotion, Sensory processing, Visual system

## Abstract

The SARS-CoV-2 pandemic has led significant social repercussions and forced people to wear face masks. Recent research has demonstrated that the human ability to infer emotions from facial configurations is significantly reduced when face masks are worn. Since the mouth region is specifically crucial for deaf people who speak sign language, the current study assessed the impact of face masks on inferring emotional facial expressions in a population of adult deaf signers. A group of 34 congenitally deaf individuals and 34 normal-hearing individuals were asked to identify happiness, sadness, fear, anger, and neutral expression on static human pictures with and without facial masks presented through smartphones. For each emotion, the percentage of correct responses with and without face masks was calculated and compared between groups. Results indicated that face masks, such as those worn due to the SARS-CoV-2 pandemic, limit the ability of people to infer emotions from facial expressions. The negative impact of face masks is significantly pronounced when deaf people have to recognize low-intensity expressions of happiness. These findings are of essential importance because difficulties in recognizing emotions from facial expressions due to mask wearing may contribute to the communication challenges experienced by the deaf community during the SARS-CoV-2 pandemic, generating feelings of frustration and exclusion.

## Introduction

Lack of hearing generates profound modifications in an individual’s interaction with the environment^1,2^. The SARS-CoV-2 pandemic has led to significant social repercussions that have further aggravated the pre-pandemic communication difficulties experienced by the deaf community^3^. Communication between humans includes verbal interactions and a wide range of non-verbal spontaneous or intentional communicative expressions and behaviours related to their emotional states. The phylogenetic primacy of visual sensory channels in primates has made these communicative signals highly relevant^4^. In particular, understanding others’ facial expressions and their related emotional signals is a crucial ability linked to healthy social and emotional interactions^5^. Impaired recognition of others’ emotional states might affect an individual’s ability to interpret social circumstances correctly and lead to maladaptive behaviours^6^. Due to the SARS-CoV-2 pandemic, other people’s facial expressions have had to be processed from behind masks, which obscure visual information from the mouth and the lower part of the face. Recent research has demonstrated that the human ability to infer emotions from facial configurations is significantly reduced when face masks are worn^7–9^. Difficulties in recognizing emotions from facial expressions due to the wearing of masks may contribute to the communication challenges experienced by the deaf community during the SARS-CoV-2 pandemic, generating feelings of frustration and exclusion^3^. Thus, the current study assessed the impact of face masks in inferring emotional facial expressions in a population of deaf adults.

Face recognition ability depends on an interplay between holistic and analytical processing^10–14^. While holistic face perception involves the combination of various facial features into an integrated whole, analytical processing refers to the perception of specific face components separately^15^. An inhibition of holistic processing can derive from a discontinuity between the upper and the lower part of the face^16^, such as that which is generated by the wearing of a face mask. This change in face processing unavoidably impacts emotion recognition and affects how people decode facial expressions^13,17,18^. Although emotional facial expressions lose their multimodal nature in deaf individuals and have to be interpreted through vision alone^19^, a growing body of literature has agreed that deaf adults do not significantly differ from hearing adults in their ability to recognize emotions when the entire face is presented^20–22^. However, it has been demonstrated that sign language, and not the lack of audition itself, influences people’s ability to recognize faces^23^ and infer emotions from facial configurations due to the lifelong focus on the mouth region^24,25^. Specifically, knowledge of sign language enhances the ability to infer happiness^26^, in which the mouth region plays a crucial part^27^. Indeed, different regions of the face, such as the eyes and mouth, are more informative for recognizing specific emotional expressions, suggesting that the respective roles of analytic and holistic information vary between types of emotion^11–14^. Typically, individuals classify expressions as happy primarily based on the mouth region, while classifications of anger, sadness, and fear are primarily based on the upper part of the face^12,16,28^.

Here, we hypothesize that the presence of face masks affects the emotion recognition abilities of deaf adults who currently speak sign language. Specifically, we expect face masks to reduce the performance of deaf people overall, similarly to the reduced performance of hearing people. Moreover, since the mouth region is crucial for inferring happiness in deaf signers, we expect that obscuring the lower part of the face with face masks specifically impairs their ability to recognize happy faces. To test these hypotheses, we asked adult deaf signers to identify facial emotions on images of faces both with and without face masks. Different intensities of facial expressions were tested to investigate mild levels of impairment and recall more realistic situations of facial configurations.

## Methods

A group of 36 congenitally deaf adults and a group of 35 normal-hearing adults were recruited from the general population. All participants were self-identified as White Italians. Two deaf participants and one hearing participant were excluded from the analyses because they were identified as outliers (i.e. their scores in at least one task differed more than two standard deviations from the group’s mean score). Thus, the remaining participants comprised 34 congenitally deaf Italian participants (mean age ± standard deviation = 60 years old ± 13.5, female = 27) and 34 Italian participants with normal hearing function (44.6 years old ± 12.98, female = 20). See Supplementary Materials for demographic details (Tables S1 and S2). All deaf participants used Italian sign language as their main means of communication. The research protocol was approved by the local ethical committee (Comitato Etico, ASL3 Genovese, Italy), and informed consent was obtained before answering the questionnaire.

**Figure 1:**
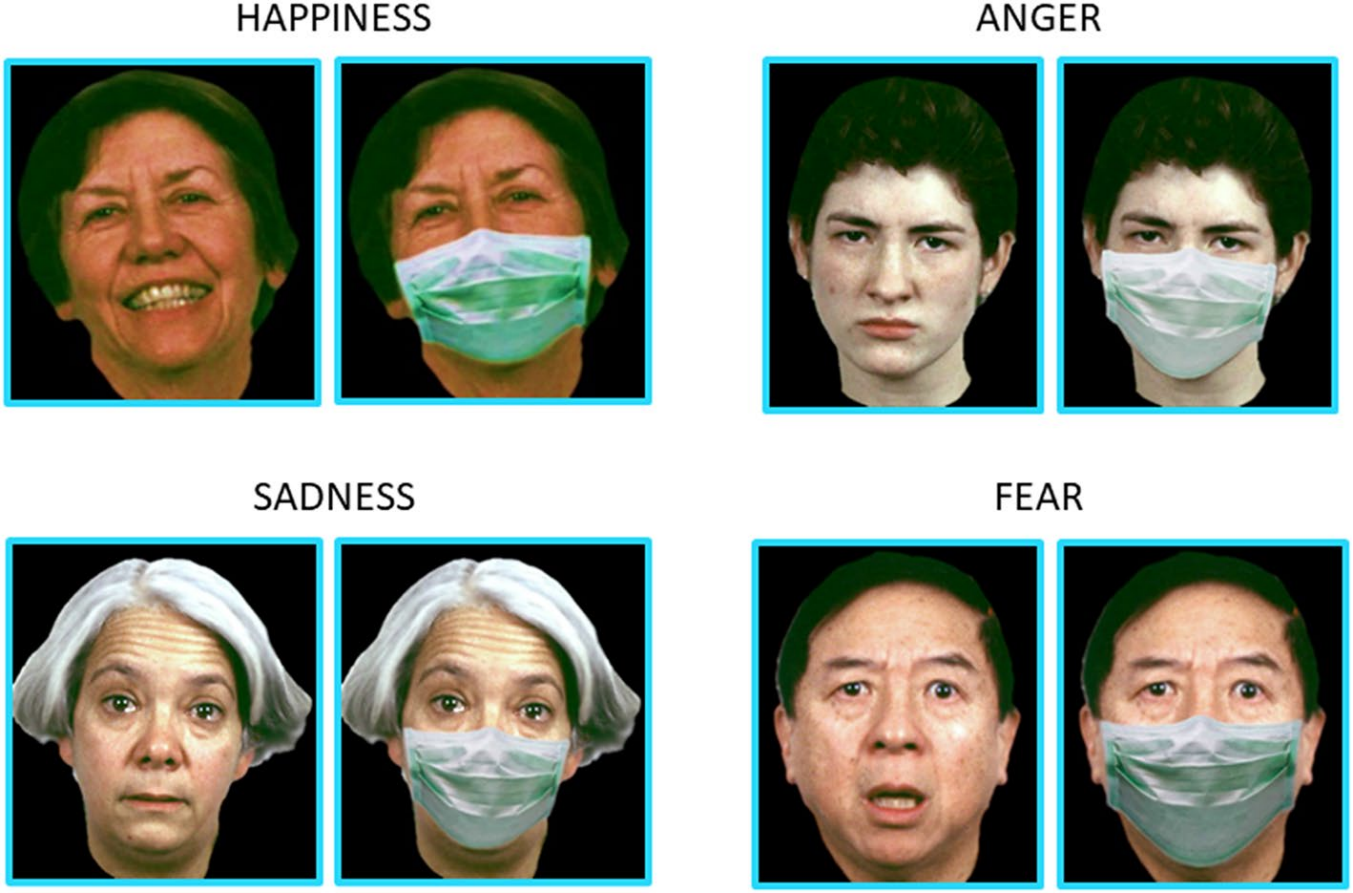
Examples of low-intensity facial configuration with and without face masks for happiness, anger, sadness, and fear. Face images were obtained with permission from the ER-40 colour emotional stimuli public database^29,30^.

**Figure 2:**
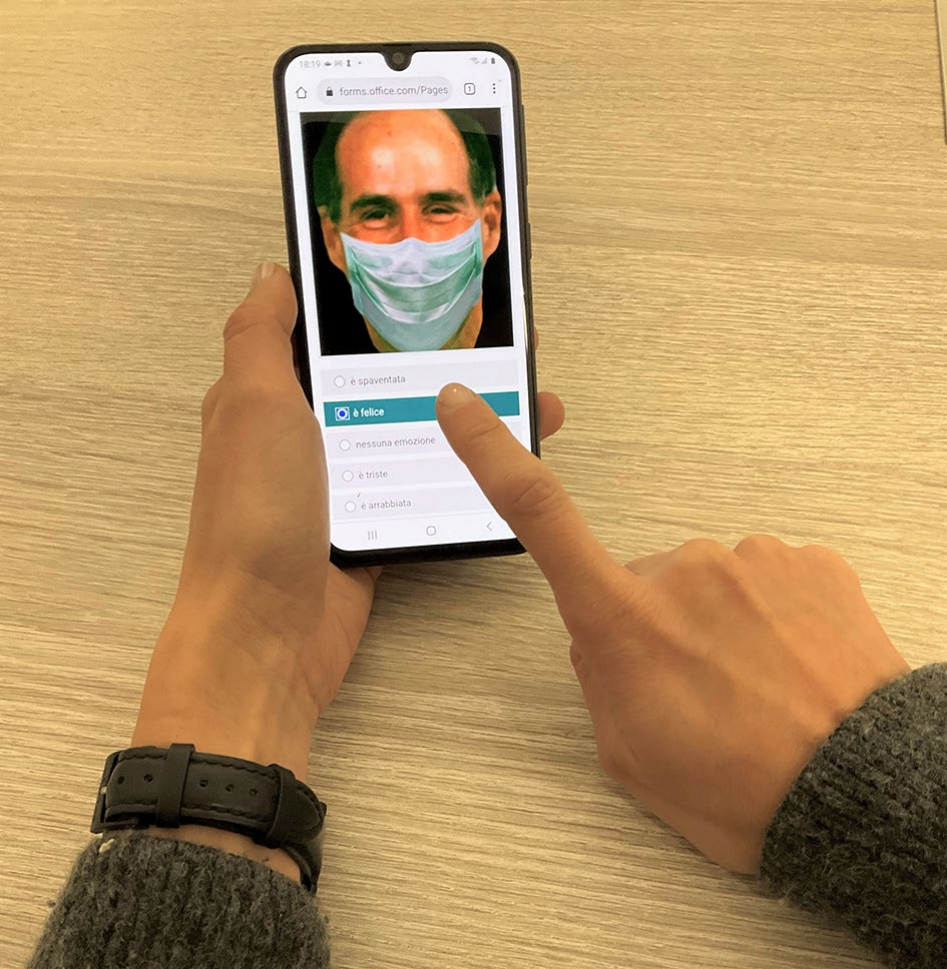
Experimental procedure. Participants were asked to identify the correct facial emotion by choosing between five possible randomized options: happy, sad, fearful, angry, and neutral. Each face was displayed on the screen of personal smartphones for as long as it took to provide the response by holding the index finger against the touch screen. Face images were obtained with permission from the ER-40 colour emotional stimuli public database^29,30^.

To investigate how face masks affect emotion recognition in deaf signers, we administered an internet-based questionnaire via smartphone that required participants to identify facial emotions on face images with and without facial masks. Specifically, we replicated the paradigm previously used to test the effects of face masks on emotion recognition during development^8^, which consisted of a standardized verbal-response test based on selecting an emotion’s label (forced choice) to describe static pictures of human facial configurations. This method was chosen to ensure the repeatability of the task and make the administration of the test easy for the subjects via smartphone in order to overcome the difficulties related to social distancing rules. The task was structured in sequential blocks, first showing a set of pictures of people wearing face masks, followed by a block of mask-free images. A total of 40 pictures of adult faces were presented in a randomized order, including four repetitions of four facial emotions (happiness, sadness, fear, and anger) at two levels of intensity (low and high) and a neutral facial expression that was presented eight times to each participant. Figure 1 shows some exemplar images for happiness, sadness, fear, and anger with a low level of intensity. The original and modified pictures were obtained from the ER-40 colour emotional stimuli database^29,30^, developed for the validated ER-40 test for facial emotion recognition^31,32^. Pictures from the original database were modified ad hoc by a web designer who created and added realistic face masks for the set of images containing masks. The ER-40 colour emotional stimuli database was selected because it is widely used, offers good construct validity and psychometric properties, and contains face images carefully obtained from people from diverse backgrounds in terms of gender, ethnicity, and age. Specifically, a facial database was originally built based on facial images from people ranging in age from 10 to 85 years (mean age ± standard deviation for men = 38.59 years old ± 14.8; women = 36.49 years old ± 16.3), from different ethnicity (91 White, 32 Black, six Asian, and 10 Hispanic). In the current study, participants were asked to identify the facial emotion by choosing from among five possible randomized options: happy, sad, fearful, angry, and neutral. Each face was displayed on the screen for as long as the response was given, and the response was selected by pressing the touch screen of a personal smartphones using an index finger. No time limits were imposed to provide an answer. Figure 2 shows a participant answering one exemplar question. To control for face mask exposure, the test was performed one year after the first lockdown ended in Italy in May 2020. Since we could not ensure complete control over the administration of the test, we provided specific written instructions to participants and carefully instructed them to perform it without any help.

For the data analysis, performance was calculated as a percentage of correct responses with and without the mask. First of all, t-tests were conducted to compare statistically the performance in each condition (i.e. mask, no mask) and group (i.e. hearing, deaf) to chance level responding (i.e. 20%, see “Supplementary Materials”). Results were corrected for multiple comparisons using a Bonferroni correction. Subsequently, performance was analysed with an ANCOVA considering mask presence (i.e. mask or no mask) and emotion (i.e. happiness, sadness, fear, anger, neutral expression) as within-subject factors, group (i.e. hearing, deaf) as between-subject factor, and age and gender as covariates. Subsequent analyses focused on each emotion separately. Thus, for each emotion, an ANOVA was run with group (i.e. hearing, deaf) as a between-subject factor and mask presence (i.e. mask, no mask) and the intensity level of emotions (i.e. low, high) as within-subject factors. Since intensity was not present as a variable for neutral faces, the omnibus ANCOVA did not involve intensity level, and for the neutral expression, we performed a two-way follow-up ANOVA considering only mask presence (i.e. mask, no mask) and group (i.e. hearing, deaf). Follow-up ANOVAs and post hoc comparisons were carried out by applying a Bonferroni correction to the results. Moreover, confusion matrices were realized to investigate the response distribution among different emotions for each group, with and without masks. For each emotion, with and without masks, analyses on type of errors were carried out with pairwise chi-squared tests comparing percentage of responses, applying a Bonferroni correction to the results.

### Ethical approval

All procedures performed in studies involving human participants were in accordance with the ethical standards of the institutional and/or national research committee and with the 1964 Helsinki declaration and its later amendments or comparable ethical standards.

### Informed consent

Informed consent was obtained from all individual participants included in the study.

## Results

**Figure 3:**
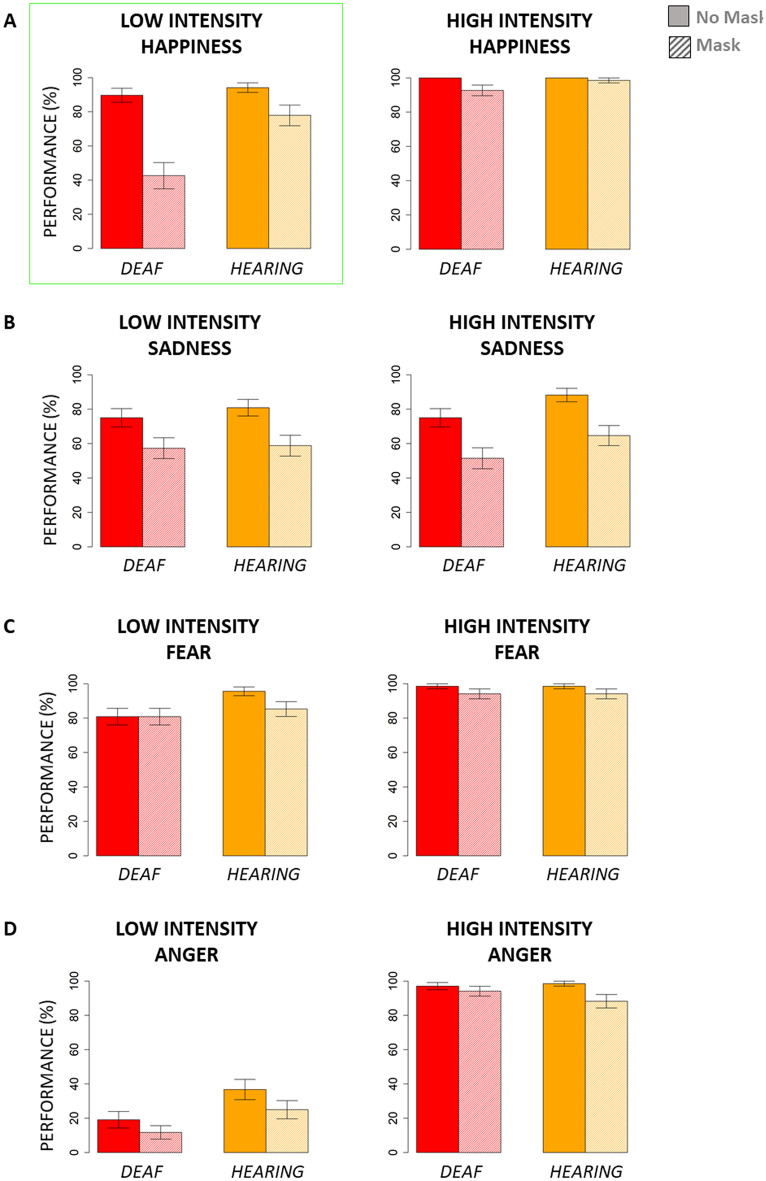
Percentage of correct responses without and with face masks in deaf and hearing people. Left: performance for images with a low level of intensity. Right: performance for images with a high level of intensity. (**A**) performance for happiness; (**B**) performance for sadness; (**C**) performance for fear; (**D**) performance for anger. The standard error of the mean (SEM) is reported.

Results showed that face masks always negatively impact the human ability to recognize emotions from facial configurations, but this is particularly true for deaf people when they have to recognize low-intensity happy images. Indeed, deaf signers’ ability to infer happiness when happy facial configurations are relatively subtle is drastically influenced by face masks (see the green rectangle in Fig. 3).

The omnibus ANCOVA demonstrated a significant main effect of mask presence (F(1, 2580) = 66.12, p < 0.01), group (F(1, 60) = 11.81, p < 0.01), and emotion (F(1, 2580) = 64.42, p < 0.01) but not age (F(1, 60) = 1.03, p > 0.5) and gender (F(1, 60) = 0.45, p > 0.5). Moreover, there was no significant interaction between age, gender, condition, emotion, and group (F(4, 2580) = 0.45, p > 0.05), allowing us to rule out age and gender having any role in the results. Instead, a significant interaction emerged from the ANCOVA between condition, emotion, and group (F(4, 2580) = 2.46, p < 0.01), creating the opportunity to analyse each emotion separately.

For happiness (Fig. 3A), the follow-up ANOVA demonstrated significant main effects of mask presence (F(1, 66) = 39.83, p < 0.01), group (F(1, 66) = 12.48, p < 0.01), and intensity (F(1, 66) = 12.48, p < 0.001). Since the interaction between these three factors was also significant (F(1, 66) = 7.26, p < 0.05), follow-up ANOVAs focused on each level of intensity to compare the performance of happy images with and without masks between the deaf and hearing groups. For happy face images with low intensity (Fig. 2 top-left), the analysis revealed a main effect of face mask presence (F_1,66_ = 41.42, p < 0.01), a main effect of group (F_1,66_ = 11.03, p < 0.01), and a strong interaction between group and condition (F_1,66_ = 9.88, p < 0.01). Through post hoc t-tests, we observed that face masks reduced the ability to label positive emotions with low intensity for both hearing (t_46.54_ = 2.43, p < 0.05) and deaf (t_50.54_ = 5.42, p < 0.01) participants, but while hearing and deaf participants performed similarly for images without face masks (t_58.3_ = -0.88, p > 0.05), deaf participants exhibited a much stronger deficit when compared to hearing participants for images with face masks (t_62.63_ = -3.62, p < 0.01). Regarding happy face images with high intensity, we found a significant effect of masks (F(1, 66) = 6.67, p < 0.05), suggesting worse performance for both groups when face masks covered the lower part of the face. Neither the main effect of group (F(1, 66) = 2.97, p > 0.05) nor the interaction between group and mask presence (F(1, 66) = 2.06, p > 0.05) were significant for high-intensity happy faces.

As for sadness (Fig. 3B), only a main effect of mask presence appeared (F(1, 66) = 53, p < 0.01), indicating participants’ overall worse performance due to face masks. All other effects were not significant (p > 0.05), including the interaction between mask presence, group, and intensity level (F(1, 66) = 0.12, p > 0.05).

For fear (Fig. 3C), the follow-up ANOVA showed again a significant main effect of mask presence (F(1, 66) = 15.65, p < 0.01), revealing a decrease in performance associated with masks. Intensity level also had a significant impact on fear perception (F(1, 66) = 4.58, p > 0.05), which indicates that low-intensity fearful faces were more difficult to recognize when compared to high-intensity ones, independent of mask presence and group, because the interaction between mask presence, group, and intensity was not significant (F(1, 66) = 1.57, p > 0.05). Other effects were not present (p > 05).

As for anger (Fig. 3D), statistical analyses showed a significant main effect of group (F(1, 66) = 5.68, p < 0.05), mask presence (F(1, 66) = 11.22, p < 0.05), and intensity (F(1, 66) = 5.68, p < 0.01). While the interaction between these three factors was not significant (F(1, 66) = 0.09, p > 0.05), the interaction between group and level of intensity was statistically significant (F(1, 66) = 7.75, p > 0.01). Post hoc t-tests revealed a worse performance for low-level angry expressions compared to highlevel ones for both deaf (t_33_ = 12.55, p < 0.01) and hearing (t_33_ = 20.45, p < 0.01) participants. However, while the two groups had similar performance for high-level angry expressions (t_61.48_ = 0.8, p > 0.05), they differed on low-level ones (t_65.04_ = −2.92, p < 0.01).

When analysing neutral expressions, we observed that the presence of masks similarly affected the performance of all participants, with no differences between groups. Indeed, a main effect of mask presence emerged from the ANOVA on performance (F_1,66_ = 10.22, p < 0.01) but not a main effect of group (F_1,66_ = 1.25, p > 0.05) or an interaction between mask presence and group (F_1,66_ = 1.35, p > 0.05).

**Figure 4:**
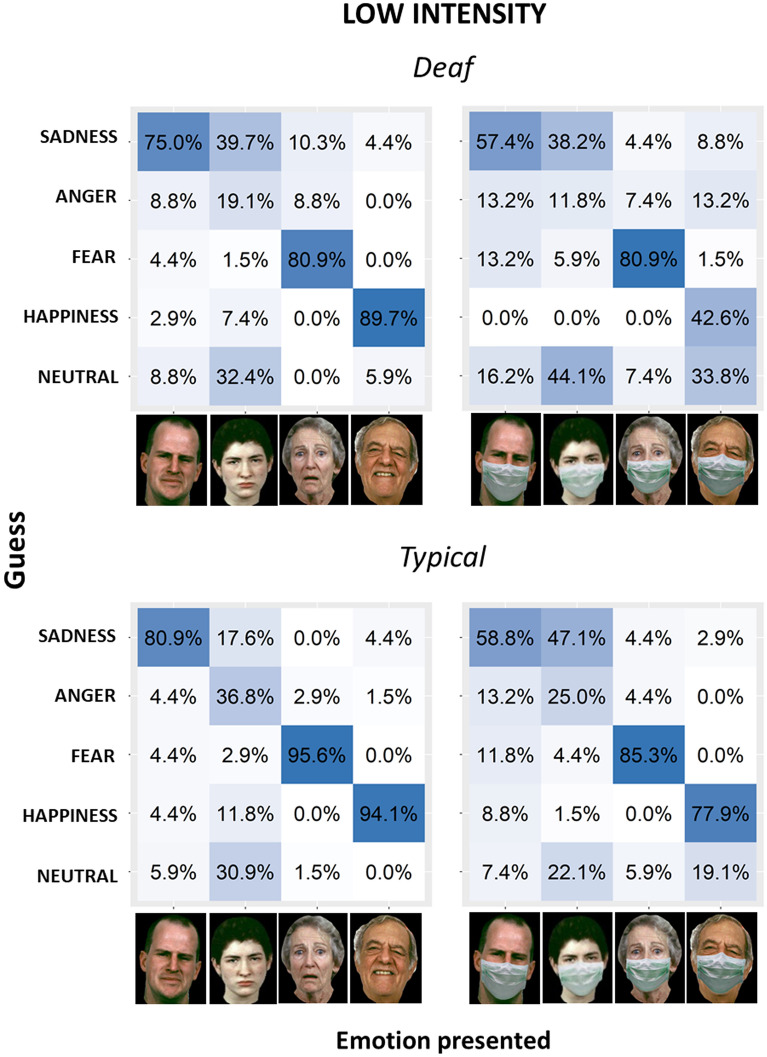
Confusion matrices for emotion inference from low-intensity facial configurations without (left) and with (right) face masks for deaf (top) and hearing (bottom) groups. The x-axis shows the presented stimuli. The y-axis shows the emotions perceived by participants. Columns report the percentage of responses for each emotion. Face images were obtained with permission from the ER-40 colour emotional stimuli public database^29,30^.

**Figure 5:**
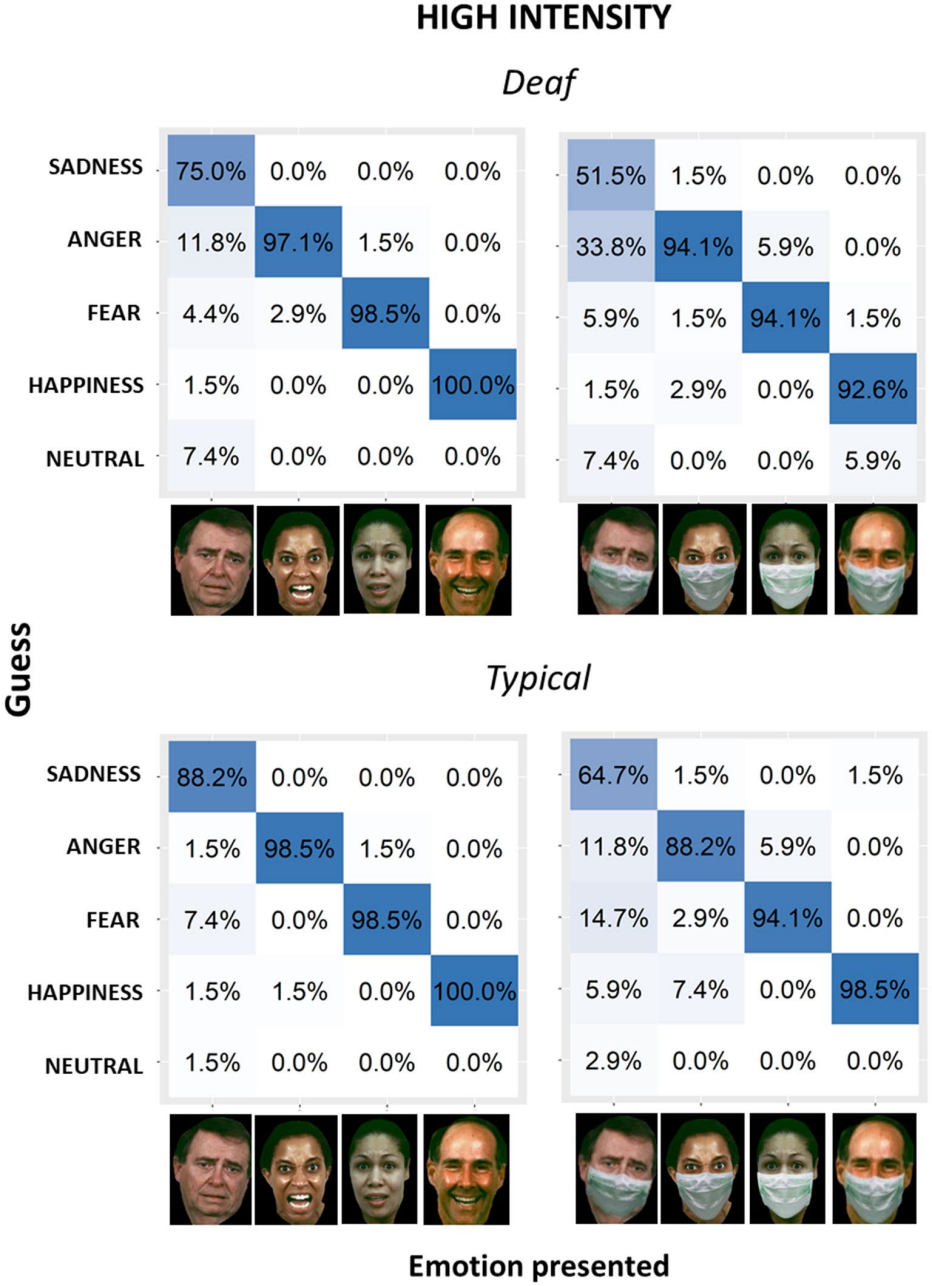
Confusion matrices for emotion inference from high-intensity facial configurations without (left) and with (right) face masks for deaf (top) and hearing (bottom) groups. The x-axis shows the presented stimuli. The y-axis shows the emotions perceived by participants. Columns report the percentage of responses for each emotion. Face images were obtained with permission from the ER-40 colour emotional stimuli public database^29,30^.

Response distributions among different emotions for each group, with and without masks, are represented in Figs. 4 and 5, which report the matrices of confusion for low level and high level of intensity, respectively. Results of pairwise chi-squared tests comparing percentage of responses for each emotion are reported in Supplementary Materials (for deaf: Tables S3, S4, S5 and S6; for hearing: Tables S7, S8, S9 and S10). All participants confused the correct emotion with other emotions more when the mask was present. For both groups, confusion increased in the low-intensity condition, and this was especially true for deaf participants. The most difficult emotion to recognize was anger (in keeping with), which was often recognized as a neutral expression or sadness, both with and without a mask, and by both groups. Specifically, hearing people did not commit any significant error when they had to recognize high-intensity emotions with or without masks (see Tables S9 and S10 in Supplementary Materials). Similarly, deaf people did not commit any significant error when they had to recognize high-intensity emotions without masks (see Table S6 in Supplementary Materials). When mask was present, deaf people easily recognized high-intensity anger, fear and happiness (see Table S5 in Supplementary Materials). Although they could identify sadness as well (percentage of response sad when images represented sadness with mask: 51.5%), sadness was sometimes confused with anger (percentage of response angry when images represented sadness with mask: 33.8%). When asked to recognize low-intensity emotions with and without masks, hearing people significantly succeeded in identifying sadness, fear and happiness (see Tables S7 and S8 in Supplementary Materials). Instead, they struggled more with anger. With masks, anger was more often interpreted as sadness, and it was not distinguished from neutral expression (percentage of response anger when images represented anger with mask: 47.1%; percentage of response sadness when images represented anger with mask: 25%; percentage of response neutral expression when images represented anger with mask: 22.1%). Instead, anger was rarely confused by hearing people with happiness and fear. Similarly, without masks, anger was often misinterpreted as sadness and neutral expression (no significant differences between percentage of response angry and percentage of response sad or neutral expression), sometimes with happiness and rarely with fear. When asked to recognize low-intensity emotions without masks, deaf people easily recognized sadness, fear and happiness (see Table S4 Supplementary Materials). For anger, instead, percentages of responses were not significantly different between anger, sadness and neutral expression, suggesting that anger was hardly distinguished from sadness and neutral expression. Actually, sadness was the most selected response for anger expression without mask (39.7%), followed by neutral expression (30.9%) and anger (17.6%); fear and happiness show the lower percentage of responses. The most interesting results about types of error are the ones about low-intensity emotions with masks for deaf people (see Table S3 Supplementary Materials), where confusion between responses increased even more. While fear and sadness could still be identified, the recognition became more difficult for the other emotions. For happiness, percentages of response happiness and neutral expression are similar (percentage of response happy when images represented happiness with mask: 42.6%; percentage of response neutral expression when images represented happiness with mask: 33.8%;), suggesting that, without seeing the mouth, happiness was often interpreted as neutral expressions. For anger, the higher percentages of response were displayed by neutral expression and sadness, while anger was less chosen, similarly to fear and happiness.

## Discussion

The use of face masks to prevent SARS-CoV-2 transmission represents a significant challenge for deaf people, posing a severe obstacle for communication^3^. Since deaf signers preferentially focus on the mouth region of the face^24,25^, here we studied whether face masks specifically affect their ability to recognize facial emotional expressions. Deaf and hearing participants were tested with a visual task to recognize emotions from facial configurations with or without a face mask. Our results indicated that face masks, such as those worn due to the SARS-CoV-2 pandemic, limit the ability of both hearing and deaf people to infer emotions from facial expressions. Moreover, the negative impact of face masks is significantly more pronounced when deaf people have to recognize the low-intensity expression of happiness.

First of all, the results align with the findings in the previous literature on the detrimental effect of face masks on people’s ability to recognize emotions from facial expressions^7,8^ and extended the deficit to the deaf population. For both groups, the masking effect was evident independent of the intensity of the facial configurations and regardless of the kinds of emotion expressed. These results support the important role of holistic processing in recognizing emotions and suggest that deaf people do not compensate with alternative visual strategies when the mouth region is covered. It has been widely demonstrated that deaf signers pay increased attention to the lower part of the face and fixate on it more than the upper part when compared to hearing people^33,34^. Thus, it is not surprising that mask wearing always compromises the performance of deaf people.

However, while the level of impairment is similar between groups for sadness, fear, anger, and neutral expression, it differs for low-intensity happy facial expressions. Specifically, when focusing on low-intensity facial configurations of happiness, the level of the impairment between deaf and hearing people changes, becoming extremely apparent for deaf people. The percentage of correct responses for deaf people when inferring happiness from low-intensity facial configurations with masks was around 40%, as compared to 75% for the hearing group. Moreover, for deaf people, performance was reduced by about 50% for subtle happy images with masks compared to those without masks. These data are impressive and suggest that when happy facial expressions are weakly expressed and hidden by a face mask, they are rarely recognized by a deaf person. Previous research can easily explain the drop in performance when deaf people are presented with weak happy facial expressions behind masks. Indeed, deaf signers preferentially focus on the lower part of the face^33,34^, and the mouth region is known to be crucial for the perception of happy faces^27^. Accordingly, Dobel et al.^26^ have demonstrated that deaf signers outperform hearing people in recognizing happy faces when expressions are very subtle, thanks to deaf signers’ preferential processing of the mouth region. Thus, by hiding the lower part of the face behind a mask, it became difficult for deaf participants to recognize low-intensity happy images.

In our study, we did not find any significant differences between the two groups for low-level happy expressions without masks, likely because our stimuli were still too clear, even in this condition, while they were subtle enough when face masks were present. Moreover, we replicated the superiority effect of happiness in both groups^35,36^. Happiness is the easiest emotion to recognize, to the extent that performance is very high for both groups when no mask is worn, reaching ceiling level when the intensity is high. We so easily recognize happiness when it is expressed at a high level that the discomfort caused by wearing a mask is minimized through high-intensity happy facial expressions, as compared to that experienced with low-intensity happy facial expressions.

As regards the other emotions, our results mostly showed support for similar recognition abilities between deaf and hearing people when no mask is worn^20–22^. In line with previous studies^23,35,37–39^, deaf and hearing people do not differ in their ability to identify sadness and fear. For both groups, fear was harder to identify when it was subtle, which is in line with previous studies using the same database of images^31^. The only difference between the groups for faces without masks was found for low-intensity angry faces, for which the performance was lower overall when compared to high-intensity angry faces and was slightly reduced for deaf people when compared with hearing people. This is in agreement again with the results of Dobel et al.^26^, showing that deaf signers performed worse than non-signers in inferring angry faces when the emotional expression was only subtle. Although some results were mixed, the eye region is crucial for recognizing anger^27,40^. Thus, deaf signers’ lifelong focus on the lower part of the face^33,34^ may be the underlying reason explaining the difference in performance we reported for low-intensity angry facial configurations. The evident low performance of all participants for weak angry faces when compared to facial expressions with pronounced anger is again in line with the results of previous studies that used the same database of images^29,31^, and it is likely a flaw intrinsic to the database. Kohler et al.^31^, for example, reported a percentage of correct responses at around 85% and 40% for anger with high and low levels of intensity, respectively.

It is important to state that this study has some limitations. Specifically, the offline testing modality unavoidably created some constraints intrinsic to the experimental context. In this regard, we could not ensure that the participants did not receive any external help or that they maintained high concentration and attention throughout the questionnaire, and we could not record their reaction times as control data. The fixed order of the blocks represents another important limitation due to the experimental procedure. Our choice of not randomizing the two blocks (i.e. faces with and without masks) was justified because we did not want the participants’ performance to be affected by having previously seen the same face but unmasked. We could have used different face images between the blocks, but this would not have allowed direct comparisons to show that face masks actually affected the participants’ ability to recognize emotions. However, we ruled out the results being driven by fatigue due to the task duration as we observed a worse performance in the first block than in the second block. Moreover, we cannot completely rule out other potential confounds, such as information about hearing loss, visual acuity, previous experience with this kind of paradigm, personality traits, or psychopathological symptoms. For instance, depression is known to influence the perception of happy faces^41^, and we cannot exclude the possibility of any of the participants suffering from this clinical condition. On the one hand, it is likely that these potential confounds similarly affected the participants’ performances in both blocks, without interfering with our main results about the effect of masks. On the other hand, these variables could have potentially impacted the participants’ performance and must be taken into account when considering this work.

To sum up, our findings enrich the current literature on deaf adults’ emotion recognition abilities and underlying visual inspection strategies. We confirmed research showing that deaf people have mostly similar abilities in this regard when compared to hearing individuals, with the exception of low-intensity anger, which deaf signers struggle more to recognize^20–22,26^. We added that face masks reduce the chances of deaf people recognizing emotional facial expressions. In addition, by observing that the possibility of recognizing happiness is drastically reduced for deaf signers when the mouth region is covered, we highlighted the specific importance that the lower part of the face has for deaf people who speak sign language. Future research should verify whether similar results characterize non-signer deaf individuals to circumscribe better the role of sign language acquisition and related visual inspection strategies. Moreover, beyond our more theoretical findings, our results are of extreme practical importance within the pandemic context in which we are living. Deaf people already have limited access to situational and contextual cues. The fact that they now have to struggle to recognize emotions, specifically happiness, due to the obstruction caused by face masks, cannot be neglected and warrants greater societal consideration. The implications of these findings are even more worrying when it comes to their impact on deaf children who experience a developmental delay in recognizing emotions^42,43^ that could potentially be aggravated during the SARS-CoV-2 pandemic by the use of face masks, thereby affecting their emotional and social development.

## Supplementary Information

Supplementary Information.
