# Supplementary material for: Face masks affect perception of happy faces in deaf people
Source: Sci Rep. 2022 Jul 20;12:12424. doi: 10.1038/s41598-022-16138-x (PMC9298172; doi:10.1038/s41598-022-16138-x)
Supplement: Supplementary file 1 — Supplementary Information. [file 41598_2022_16138_MOESM1_ESM.pdf]

SUPPLEMENTARY MATERIALS

Face masks affect perception of happy faces in deaf people

Amadeo Maria Bianca[1,2*], Escelsior Andrea[2,3,4*], Amore Mario[2,3,4], Serafini Gianluca[2,3,4], Pereira da Silva Beatriz[1,2,3,4], Gori Monica[1,2]

[1] U-VIP Unit for Visually Impaired People, Fondazione Istituto Italiano di Tecnologia, Genoa, Italy

[2] Applied Neurosciences for Technological Advances in Rehabilitation Systems (ANTARES) Joint Lab, Clinica Psichiatrica ed SPDC, Largo Rosanna Benzi, 10 - 16132 Genova (GE)

[3] Department of Neuroscience, Rehabilitation, Ophthalmology, Genetics, Maternal and Child Health (DINOGMI), Section of Psychiatry, University of Genoa, Genoa, Italy

[4] IRCCS Ospedale Policlinico San Martino, Genoa, Italy

*First authors: Maria Bianca Amadeo, Andrea Escelsior

Corresponding author: Maria Bianca Amadeo; email: mariabianca.amadeo@iit.it; address: U-VIP Unit for Visually Impaired People, Fondazione Istituto Italiano di Tecnologia, via Enrico Melen 83, 16152, Genoa, Italy; phone: +39 0108172232

Results

Demographic details of deaf and hearing participants are shown in Table S1 and Table S2 respectively. A preliminary analysis revealed that the ability of inferring emotions from facial configurations was, for all participants, significantly above-chance level both without face masks (for Hearing: $t_{33} = 49.1$, $p < 0.001$; for Deaf: $t_{33} = 31.98$, $p < 0.001$) and with face masks (for Hearing: $t_{33} = 29.87$, $p < 0.001$; for Deaf: $t_{33} = 25.86$, $p < 0.001$).

ID	GROUP	AGE	GENDER
S01	Deaf	18	F
S02	Deaf	18	F
S03	Deaf	39	F
S04	Deaf	48	F
S05	Deaf	52	F
S06	Deaf	58	F
S07	Deaf	63	F
S08	Deaf	63	F
S09	Deaf	63	F
S10	Deaf	65	F
S11	Deaf	65	F
S12	Deaf	66	F
S13	Deaf	66	F
S14	Deaf	67	F
S15	Deaf	67	F
S16	Deaf	68	F
S17	Deaf	68	F
S18	Deaf	70	F
S19	Deaf	70	F
S20	Deaf	72	F
S21	Deaf	44	M
S22	Deaf	49	M
S23	Deaf	55	M
S24	Deaf	56	M
S25	Deaf	56	M
S26	Deaf	60	M
S27	Deaf	68	M
S28	Deaf	68	M
S29	Deaf	69	M
S30	Deaf	69	M
S31	Deaf	70	M
S32	Deaf	70	M
S33	Deaf	70	M
S34	Deaf	70	M

Table S1 Demographic details of deaf people.

ID	GROUP	AGE	GENDER
S35	Typical	21	F
S36	Typical	30	F
S37	Typical	32	F
S38	Typical	32	F
S39	Typical	34	F
S40	Typical	35	F
S41	Typical	36	F
S42	Typical	38	F
S43	Typical	40	F
S44	Typical	42	F
S45	Typical	42	F
S46	Typical	43	F
S47	Typical	45	F
S48	Typical	45	F
S49	Typical	46	F
S50	Typical	46	F
S51	Typical	46	F
S52	Typical	46	F
S53	Typical	46	F
S54	Typical	48	F
S55	Typical	51	F
S56	Typical	52	F
S57	Typical	60	F
S58	Typical	60	F
S59	Typical	65	F
S60	Typical	65	F
S61	Typical	67	F
S62	Typical	21	M
S63	Typical	25	M
S64	Typical	30	M
S65	Typical	46	M
S66	Typical	49	M
S67	Typical	65	M
S68	Typical	69	M

Table S2 Demographic details of hearing people.

Emotion presented	*Guess comparisons*			*X-squared*	*p*	*Significance level*
SADNESS	Sadness	Vs.	Anger	18.7	< 0.001	**
	Sadness	Vs.	Fear	18.7	< 0.001	**
	Sadness	Vs.	Happiness	39	< 0.001	**
	Sadness	Vs.	Neutral	15.7	< 0.001	**
	Anger	Vs.	Fear	0.0001	0.9	
	Anger	Vs.	Happiness	9	0.03	*
	Anger	Vs.	Neutral	0.2	0.2	
	Fear	Vs.	Happiness	9	0.03	*
	Fear	Vs.	Neutral	0.2	0.2	
	Happiness	Vs.	Neutral	11	< 0.001	**
ANGER	Sadness	Vs.	Anger	9.5	0.02	*
	Sadness	Vs.	Fear	16.1	< 0.001	**
	Sadness	Vs.	Happiness	26	< 0.001	**
	Sadness	Vs.	Neutral	0.3	0.2	
	Anger	Vs.	Fear	1.3	0.2	
	Anger	Vs.	Happiness	8	0.05	
	Anger	Vs.	Neutral	12.7	0.003	**
	Fear	Vs.	Happiness	4	0.2	
	Fear	Vs.	Neutral	19.9	< 0.001	**
	Happiness	Vs.	Neutral	30	< 0.001	**
FEAR	Sadness	Vs.	Anger	0.5	0.2	
	Sadness	Vs.	Fear	46.6	< 0.001	**
	Sadness	Vs.	Happiness	3	0.2	
	Sadness	Vs.	Neutral	0.5	0.2	
	Anger	Vs.	Fear	41.6	< 0.001	**
	Anger	Vs.	Happiness	5	0.2	
	Anger	Vs.	Neutral	0.0001	0.2	
	Fear	Vs.	Happiness	55	< 0.001	**
	Fear	Vs.	Neutral	41.7	< 0.001	**
	Happiness	Vs.	Neutral	5	0.2	
HAPPINESS	Sadness	Vs.	Anger	0.6	0.2	
	Sadness	Vs.	Fear	3.6	0.2	
	Sadness	Vs.	Happiness	15.11	0.001	*
	Sadness	Vs.	Neutral	10	0.01	*
	Anger	Vs.	Fear	6.4	0.1	
	Anger	Vs.	Happiness	10.5	0.01	*
	Anger	Vs.	Neutral	6.1	0.1	
	Fear	Vs.	Happiness	26.1	< 0.001	**
	Fear	Vs.	Neutral	20.2	<0.0001	**
	Happiness	Vs.	Neutral	0.7	0.2	

Table S3 Analyses on confusion matrix of deaf individuals for low-intensity emotions with masks.

Emotion presented	*Guess comparisons*			*X-squared*	*p*	*Significance level*
SADNESS	Sadness	Vs.	Anger	35.5	< 0.001	**
	Sadness	Vs.	Fear	42.7	< 0.001	**
	Sadness	Vs.	Happiness	45.3	< 0.001	**
	Sadness	Vs.	Neutral	35.5	< 0.001	**
	Anger	Vs.	Fear	1	0.9	
	Anger	Vs.	Happiness	2	0.9	
	Anger	Vs.	Neutral	0	0.9	
	Fear	Vs.	Happiness	0.2	0.9	
	Fear	Vs.	Neutral	1	0.9	
	Happiness	Vs.	Neutral	2	0.9	
ANGER	Sadness	Vs.	Anger	4.9	0.3	
	Sadness	Vs.	Fear	24.1	< 0.001	**
	Sadness	Vs.	Happiness	15.1	0.001	*
	Sadness	Vs.	Neutral	0.5	0.9	
	Anger	Vs.	Fear	10.28	0.01	*
	Anger	Vs.	Happiness	3.6	0.6	
	Anger	Vs.	Neutral	2.3	0.9	
	Fear	Vs.	Happiness	2.7	0.9	
	Fear	Vs.	Neutral	19.2	< 0.001	**
	Happiness	Vs.	Neutral	10.7	0.01	*
FEAR	Sadness	Vs.	Anger	0.08	0.9	
	Sadness	Vs.	Fear	37.2	< 0.001	**
	Sadness	Vs.	Happiness	7	0.08	
	Sadness	Vs.	Neutral	7	0.08	
	Anger	Vs.	Fear	39.4	< 0.001	**
	Anger	Vs.	Happiness	6	0.1	
	Anger	Vs.	Neutral	6	0.1	
	Fear	Vs.	Happiness	55	< 0.001	**
	Fear	Vs.	Neutral	55	< 0.001	**
	Happiness	Vs.	Neutral	-	-	
HAPPINESS	Sadness	Vs.	Anger	3	0.8	
	Sadness	Vs.	Fear	3	0.8	
	Sadness	Vs.	Happiness	52.6	< 0.001	**
	Sadness	Vs.	Neutral	0.1	0.9	
	Anger	Vs.	Fear	-	-	
	Anger	Vs.	Happiness	61	< 0.001	**
	Anger	Vs.	Neutral	4	0.5	
	Fear	Vs.	Happiness	61	< 0.001	**
	Fear	Vs.	Neutral	4	0.5	
	Happiness	Vs.	Neutral	50	< 0.001	**

Table S4 Analyses on confusion matrix of deaf individuals for low-intensity emotions without masks. When both the percentages of response were zero and chi-squared tests were not possible.

Emotion presented	*Guess comparisons*			*X-squared*	*p*	*Significance level*
SADNESS	Sadness	Vs.	Anger	2.5	0.2	
	Sadness	Vs.	Fear	24.6	< 0.001	**
	Sadness	Vs.	Happiness	32.1	< 0.001	**
	Sadness	Vs.	Neutral	22.5	< 0.001	**
	Anger	Vs.	Fear	13.4	0.003	*
	Anger	Vs.	Happiness	20.6	< 0.001	**
	Anger	Vs.	Neutral	11.6	0.007	*
	Fear	Vs.	Happiness	1.8	0.2	
	Fear	Vs.	Neutral	0.1	0.2	
	Happiness	Vs.	Neutral	2.7	0.2	
ANGER	Sadness	Vs.	Anger	61.1	< 0.001	**
	Sadness	Vs.	Fear	0.0001	0.9	
	Sadness	Vs.	Happiness	0.3	0.9	
	Sadness	Vs.	Neutral	1	0.9	
	Anger	Vs.	Fear	61.1	< 0.001	**
	Anger	Vs.	Happiness	58.2	< 0.001	**
	Anger	Vs.	Neutral	64	< 0.001	**
	Fear	Vs.	Happiness	0.3	0.9	
	Fear	Vs.	Neutral	1	0.9	
	Happiness	Vs.	Neutral	2	0.9	
FEAR	Sadness	Vs.	Anger	4	0.4	
	Sadness	Vs.	Fear	64	< 0.001	**
	Sadness	Vs.	Happiness	-	-	
	Sadness	Vs.	Neutral	-	-	
	Anger	Vs.	Fear	52.9	< 0.001	**
	Anger	Vs.	Happiness	4	0.4	
	Anger	Vs.	Neutral	4	0.4	
	Fear	Vs.	Happiness	64	< 0.001	**
	Fear	Vs.	Neutral	64	< 0.001	**
	Happiness	Vs.	Neutral	-	-	-
HAPPINESS	Sadness	Vs.	Anger	-	-	
	Sadness	Vs.	Fear	1	0.9	
	Sadness	Vs.	Happiness	63	< 0.001	**
	Sadness	Vs.	Neutral	4	0.4	
	Anger	Vs.	Fear	1	0.9	
	Anger	Vs.	Happiness	63	< 0.001	**
	Anger	Vs.	Neutral	4	0.4	
	Fear	Vs.	Happiness	60.1	< 0.001	**
	Fear	Vs.	Neutral	1.8	0.9	
	Happiness	Vs.	Neutral	52	< 0.001	**

Table S5 Analyses on confusion matrix of deaf individuals for high-intensity emotion with masks. When both the percentages of response were zero and chi-squared tests were not possible.

Emotion presented	*Guess comparisons*			*X-squared*	*p*	*Significance level*
SADNESS	Sadness	Vs.	Anger	31.3	< 0.001	**
	Sadness	Vs.	Fear	42.7	< 0.001	**
	Sadness	Vs.	Happiness	48.1	< 0.001	**
	Sadness	Vs.	Neutral	37.8	< 0.001	**
	Anger	Vs.	Fear	2.3	0.9	
	Anger	Vs.	Happiness	5.4	0.2	
	Anger	Vs.	Neutral	0.7	0.9	
	Fear	Vs.	Happiness	1	0.9	
	Fear	Vs.	Neutral	0.5	0.9	
	Happiness	Vs.	Neutral	2.7	0.9	
ANGER	Sadness	Vs.	Anger	66	< 0.001	**
	Sadness	Vs.	Fear	2	0.9	
	Sadness	Vs.	Happiness	-	-	
	Sadness	Vs.	Neutral	-	-	
	Anger	Vs.	Fear	60.2	< 0.001	**
	Anger	Vs.	Happiness	66	< 0.001	**
	Anger	Vs.	Neutral	66	< 0.001	**
	Fear	Vs.	Happiness	2	0.9	
	Fear	Vs.	Neutral	2	0.9	
	Happiness	Vs.	Neutral	-	-	
FEAR	Sadness	Vs.	Anger	1	0.9	
	Sadness	Vs.	Fear	67	< 0.001	**
	Sadness	Vs.	Happiness	-	-	
	Sadness	Vs.	Neutral	-	-	
	Anger	Vs.	Fear	64	< 0.001	**
	Anger	Vs.	Happiness	1	0.9	
	Anger	Vs.	Neutral	1	0.9	
	Fear	Vs.	Happiness	67	< 0.001	**
	Fear	Vs.	Neutral	67	< 0.001	**
	Happiness	Vs.	Neutral	-	-	
HAPPINESS	Sadness	Vs.	Anger	-	-	
	Sadness	Vs.	Fear	-	-	
	Sadness	Vs.	Happiness	68	< 0.001	**
	Sadness	Vs.	Neutral	-	-	
	Anger	Vs.	Fear	-	-	
	Anger	Vs.	Happiness	68	< 0.001	**
	Anger	Vs.	Neutral	-	-	
	Fear	Vs.	Happiness	68	< 0.001	**
	Fear	Vs.	Neutral	-	-	
	Happiness	Vs.	Neutral		< 0.001	**

Table S6 Analyses on confusion matrix of deaf individuals for high-intensity emotion without masks. When both the percentages of response were zero and chi-squared tests were not possible.

Emotion presented	*Guess comparisons*			*X-squared*	*p*	*Significance level*
SADNESS	Sadness	Vs.	Anger	19.6	< 0.001	**
	Sadness	Vs.	Fear	21.3	< 0.001	**
	Sadness	Vs.	Happiness	25.1	< 0.001	**
	Sadness	Vs.	Neutral	27.2	< 0.001	**
	Anger	Vs.	Fear	0.06	0.9	
	Anger	Vs.	Happiness	0.6	0.9	
	Anger	Vs.	Neutral	1.1	0.9	
	Fear	Vs.	Happiness	0.3	0.9	
	Fear	Vs.	Neutral	0.7	0.9	
	Happiness	Vs.	Neutral	0.1	0.9	
ANGER	Sadness	Vs.	Anger	4.6		
	Sadness	Vs.	Fear	24	< 0.001	**
	Sadness	Vs.	Happiness	29.1	< 0.001	**
	Sadness	Vs.	Neutral	6.1	0.1	
	Anger	Vs.	Fear	9.8	0.01	*
	Anger	Vs.	Happiness	14.2	0.001	*
	Anger	Vs.	Neutral	0.1	0.9	
	Fear	Vs.	Happiness	1	0.9	
	Fear	Vs.	Neutral	8	0.05	
	Happiness	Vs.	Neutral	12.3	< 0.001	**
FEAR	Sadness	Vs.	Anger	0	0.9	
	Sadness	Vs.	Fear	50	< 0.001	**
	Sadness	Vs.	Happiness	3	0.8	
	Sadness	Vs.	Neutral	0.1	0.9	
	Anger	Vs.	Fear	50	< 0.001	**
	Anger	Vs.	Happiness	3	0.8	
	Anger	Vs.	Neutral	0.1	0.9	
	Fear	Vs.	Happiness	58	< 0.001	**
	Fear	Vs.	Neutral	47	< 0.001	**
	Happiness	Vs.	Neutral	4	0.4	
HAPPINESS	Sadness	Vs.	Anger	2	0.9	
	Sadness	Vs.	Fear	2	0.9	
	Sadness	Vs.	Happiness	47	< 0.001	**
	Sadness	Vs.	Neutral	8.1	0.05	
	Anger	Vs.	Fear	-	-	
	Anger	Vs.	Happiness	53	< 0.001	**
	Anger	Vs.	Neutral	13	0.003	*
	Fear	Vs.	Happiness	53	< 0.001	**
	Fear	Vs.	Neutral	13	0.003	*
	Happiness	Vs.	Neutral	24.2	< 0.001	**

Table S7 Analyses on confusion matrix of hearing individuals for low-intensity emotions with masks. When both the percentages of response were zero and chi-squared tests were not possible.

Emotion presented	*Guess comparisons*			*X-squared*	*p*	*Significance level*
SADNESS	Sadness	Vs.	Anger	46.6	< 0.001	**
	Sadness	Vs.	Fear	46.6	< 0.001	**
	Sadness	Vs.	Happiness	46.6	< 0.001	**
	Sadness	Vs.	Neutral	44.1	< 0.001	**
	Anger	Vs.	Fear	0	0.9	
	Anger	Vs.	Happiness	0	0.9	
	Anger	Vs.	Neutral	0.1	0.9	
	Fear	Vs.	Happiness	0	0.9	
	Fear	Vs.	Neutral	0.1	0.9	
	Happiness	Vs.	Neutral	0.1	0.9	
ANGER	Sadness	Vs.	Anger	4.6	0.3	
	Sadness	Vs.	Fear	7.1	0.008	*
	Sadness	Vs.	Happiness	0.8	0.9	
	Sadness	Vs.	Neutral	2.5	0.9	
	Anger	Vs.	Fear	19.6	< 0.001	**
	Anger	Vs.	Happiness	8.8	0.003	*
	Anger	Vs.	Neutral	0.3	0.9	
	Fear	Vs.	Happiness	3.6	0.6	
	Fear	Vs.	Neutral	15.7	< 0.001	**
	Happiness	Vs.	Neutral	5.9	0.2	
FEAR	Sadness	Vs.	Anger	2	0.9	
	Sadness	Vs.	Fear	65	< 0.001	**
	Sadness	Vs.	Happiness	-	-	
	Sadness	Vs.	Neutral	1	0.9	
	Anger	Vs.	Fear	59.2	< 0.001	**
	Anger	Vs.	Happiness	2	0.9	
	Anger	Vs.	Neutral	0.3	0.9	
	Fear	Vs.	Happiness	65	< 0.001	**
	Fear	Vs.	Neutral	62.1	< 0.001	**
	Happiness	Vs.	Neutral	1	0.9	
HAPPINESS	Sadness	Vs.	Anger	1	0.9	
	Sadness	Vs.	Fear	3	0.8	
	Sadness	Vs.	Happiness	55.5	< 0.001	**
	Sadness	Vs.	Neutral	3	0.8	
	Anger	Vs.	Fear	1	0.9	
	Anger	Vs.	Happiness	61.1	< 0.001	**
	Anger	Vs.	Neutral	1	0.9	
	Fear	Vs.	Happiness	64	< 0.001	**
	Fear	Vs.	Neutral	-	-	
	Happiness	Vs.	Neutral	64	< 0.001	**

Table S8 Analyses on confusion matrix of hearing individuals for low-intensity emotion without masks. When both the percentages of response were zero and chi-squared tests were not possible.

Emotion presented	*Guess comparisons*			*X-squared*	*p*	*Significance level*
SADNESS	Sadness	Vs.	Anger	24.9	< 0.001	**
	Sadness	Vs.	Fear	21.4	< 0.001	**
	Sadness	Vs.	Happiness	33.3	< 0.001	**
	Sadness	Vs.	Neutral	38.3	< 0.001	**
	Anger	Vs.	Fear	0.2	0.9	
	Anger	Vs.	Happiness	1.3	0.9	
	Anger	Vs.	Neutral	3.6	0.6	
	Fear	Vs.	Happiness	2.6	0.9	
	Fear	Vs.	Neutral	5.3	0.2	
	Happiness	Vs.	Neutral	0.6	0.9	
ANGER	Sadness	Vs.	Anger	67	< 0.001	**
	Sadness	Vs.	Fear	0.3	0.9	
	Sadness	Vs.	Happiness	2.7	0.9	
	Sadness	Vs.	Neutral	1	0.9	
	Anger	Vs.	Fear	54.3	< 0.001	**
	Anger	Vs.	Happiness	46.5	< 0.001	**
	Anger	Vs.	Neutral	60	< 0.001	**
	Fear	Vs.	Happiness	1.3	0.9	
	Fear	Vs.	Neutral	2	0.9	
	Happiness	Vs.	Neutral	5	0.3	
FEAR	Sadness	Vs.	Anger	4	0.5	
	Sadness	Vs.	Fear	64	< 0.001	**
	Sadness	Vs.	Happiness	-	-	
	Sadness	Vs.	Neutral	-	-	
	Anger	Vs.	Fear	53	< 0.001	**
	Anger	Vs.	Happiness	4	0.5	
	Anger	Vs.	Neutral	4	0.5	
	Fear	Vs.	Happiness	64	< 0.001	**
	Fear	Vs.	Neutral	64	< 0.001	**
	Happiness	Vs.	Neutral	-	-	
HAPPINESS	Sadness	Vs.	Anger	1	0.9	
	Sadness	Vs.	Fear	1	0.9	
	Sadness	Vs.	Happiness	64.1	< 0.001	**
	Sadness	Vs.	Neutral	1	0.9	
	Anger	Vs.	Fear	-	-	
	Anger	Vs.	Happiness	67	< 0.001	**
	Anger	Vs.	Neutral	-	-	
	Fear	Vs.	Happiness	67	< 0.001	**
	Fear	Vs.	Neutral	-	-	
	Happiness	Vs.	Neutral	67	< 0.001	**

Table S9 Analyses on confusion matrix of hearing individuals for high-intensity emotion with masks. When both the percentages of response were zero and chi-squared tests were not possible.

Emotion presented	*Guess comparisons*			*X-squared*	*p*	*Significance level*
SADNESS	Sadness	Vs.	Anger	57.1	< 0.001	**
	Sadness	Vs.	Fear	44.2	< 0.001	**
	Sadness	Vs.	Happiness	57.1	< 0.001	**
	Sadness	Vs.	Neutral	60	< 0.001	**
	Anger	Vs.	Fear	3.6	‘0.6	
	Anger	Vs.	Happiness	0	0.9	
	Anger	Vs.	Neutral	1	0.9	
	Fear	Vs.	Happiness	3.6	0.6	
	Fear	Vs.	Neutral	6	0.1	
	Happiness	Vs.	Neutral	1	0.9	
ANGER	Sadness	Vs.	Anger	66	< 0.001	**
	Sadness	Vs.	Fear	-	-	
	Sadness	Vs.	Happiness	1	0.9	
	Sadness	Vs.	Neutral	-	-	
	Anger	Vs.	Fear	66	< 0.001	**
	Anger	Vs.	Happiness	63.1	< 0.001	**
	Anger	Vs.	Neutral	63.1	< 0.001	**
	Fear	Vs.	Happiness	1	0.9	
	Fear	Vs.	Neutral	-	-	
	Happiness	Vs.	Neutral	0	0.9	
FEAR	Sadness	Vs.	Anger	1	0.9	
	Sadness	Vs.	Fear	67	< 0.001	**
	Sadness	Vs.	Happiness	-	-	
	Sadness	Vs.	Neutral	-	-	
	Anger	Vs.	Fear	64.1	< 0.001	**
	Anger	Vs.	Happiness	1	0.9	
	Anger	Vs.	Neutral	1	0.9	
	Fear	Vs.	Happiness	67	< 0.001	**
	Fear	Vs.	Neutral	67	< 0.001	**
	Happiness	Vs.	Neutral	-	-	
HAPPINESS	Sadness	Vs.	Anger	-	-	
	Sadness	Vs.	Fear	-	-	
	Sadness	Vs.	Happiness	68	< 0.001	**
	Sadness	Vs.	Neutral	-	-	
	Anger	Vs.	Fear	-	-	
	Anger	Vs.	Happiness	68	< 0.001	**
	Anger	Vs.	Neutral	-	-	
	Fear	Vs.	Happiness	68	< 0.001	**
	Fear	Vs.	Neutral	-	-	
	Happiness	Vs.	Neutral	68	< 0.001	**

Table S10 Analyses on confusion matrix of hearing individuals for high-intensity emotion without masks. When both the percentages of response were zero and chi-squared tests were not possible.
